# Markers of Cardiovascular Disease among Adults Exposed to Smoke from the Hazelwood Coal Mine Fire

**DOI:** 10.3390/ijerph18041587

**Published:** 2021-02-08

**Authors:** Juliana Betts, Elizabeth M. Dewar, Dion Stub, Caroline X. Gao, David W. Brown, Jillian F. Ikin, Berihun M. Zeleke, Sinjini Biswas, Michael J. Abramson, Danny Liew

**Affiliations:** 1 School of Public Health and Preventive Medicine, Monash University, Melbourne, VIC 3004, Australia; juliana.betts@gmail.com (J.B.); dion.stub@monash.edu (D.S.); caroline.gao@monash.edu (C.X.G.); david.brown@monash.edu (D.W.B.); jill.blackman@monash.edu (J.F.I.); berihun.zeleke@monash.edu (B.M.Z.); sinjini@gmail.com (S.B.); danny.liew@monash.edu (D.L.); 2 Department of Cardiology, Alfred Health, Melbourne, VIC 3004, Australia; l.dewar@alfred.org.au; 3 Baker Heart and Diabetes Institute, Melbourne, VIC 3004, Australia

**Keywords:** cardiac risk factors and prevention, inflammatory markers, epidemiology, coronary artery disease

## Abstract

Little research has examined the effects of high concentration, medium-duration smoke exposure on cardiovascular health. We investigated whether six weeks of exposure to smoke from the 2014 Hazelwood coal mine fire in Victoria (Australia), was associated with long-term clinical or subclinical cardiovascular disease approximately four years later, in adult residents of the towns of Morwell (exposed, *n* = 336) and Sale (unexposed, *n* = 162). The primary outcome was serum high sensitivity (hs) C-reactive protein (CRP). Blood pressure, electrocardiogram, flow mediated dilatation and serum levels of hs-troponin, N-terminal pro B-type natriuretic peptide and lipids were secondary outcomes. There was no significant difference in weighted median hsCRP levels between exposed and unexposed participants (1.9 mg/L vs. 1.6 mg/L, *p* = 0.273). Other outcomes were comparable between the groups. hsCRP was associated in a predictable manner with current smoking, obesity and use of lipid-lowering therapy. Four years after a 6-week coal mine fire, this study found no association between smoke exposure and markers of clinical or subclinical cardiovascular disease in exposed adults.

## 1. Introduction

Exposure to long-term ambient air pollution, especially fine particulate matter with an aerodynamic diameter < 2.5 µm (PM_2.5_), is associated with adverse cardiovascular health consequences such as ischaemic heart disease (IHD), heart failure and stroke [1,2]. Similarly, acute increments in PM_2.5_ have been associated with increased cardiovascular-related mortality and hospital admissions [3,4]. In 2015 it was estimated that over 2 million deaths and 50 million disability-adjusted life-years resulted from pollution-attributable cardiovascular disease (CVD)—evidence of the significant additional disease burden imposed by this novel risk factor [5]. Studies of long-term ambient PM_2.5_ exposure are often based in urban areas and draw upon concentrations from sources such as traffic emissions, wood heaters and industry [6,7]. Some studies of acute changes in PM_2.5_ investigate daily changes in the same urban settings, whilst others investigate the effects of discrete high concentration particulate generating events, such as landscape fires which might dissipate in hours or days [8,9]. However, there is little evidence available in regard to the long-term health effects of high concentration, medium-duration PM_2.5_ exposures. During February and March 2014 a bushfire in the Latrobe Valley (Victoria, Australia) took hold in the Hazelwood power station’s open-cut brown coal mine. It was estimated that 140 kT of coal burned over the 6-week course of the fire, severely reducing local air quality, particularly in the adjacent town of Morwell [10,11]. Uncertainty about the expected duration of the fire, and limited research on coal-mine fire emission factors and the adverse health effects of medium duration smoke exposure, hampered authorities’ efforts to formulate appropriate public health protection messages [12]. In order to learn from the fire and monitor the long-term health effects, the Victorian Department of Health commissioned the Hazelwood Health Study (HHS); a program of work comprising several research streams over at least a ten year period [13].

The Exposure Assessment Stream of the HHS, led by the Commonwealth Scientific and Industrial Research Organisation (CSIRO) Oceans and Atmosphere, modelled the smoke-related PM_2.5_ distribution in the vicinity of the fire [10,11]. The model predicted hourly PM_2.5_ concentrations as high as 3730 μg/m^3^ in the southern part of Morwell in the first days of the smoke event. The Australia National Environment Protection Measure (NEPM) air quality standard for PM_2.5_ is 25 μg/m^3^ as a 24-h average [14]. CSIRO’s modelling showed that NEPM standards were exceeded on 23 days in the southern part of Morwell and 12 days in the eastern part [11].

The Hazelinks Stream of the HHS found a 62% increase in risk of death from cardiovascular conditions in Morwell (95% CI 25–110%) evident in the 6-month period following the smoke event; equivalent to 26 attributable cases [15]. Three years after the fire, the Early Life Follow Up Stream of the HHS showed that locally residing infants aged up to 2 years at the time of smoke exposure had increases in vascular stiffness, and that children whose mothers smoked appeared to be more susceptible to the adverse effects of the mine fire PM_2.5_ on cardiovascular health [16]. Using retrospective data linkage, Hazelinks found that a 10 µg/m^3^ increase in individual-level mean PM_2·5_ exposure was associated with increased risk of cardiovascular related ambulance attendances (Adj HR:1.13; 95% CI 1.01, 1.28) among participants from the HHS Adult Survey stream during the 3.5 years after the fire [17].

C-reactive protein (CRP) is an acute phase reactant released by the liver and a highly sensitive marker of inflammation which has been associated with chronic exposure to PM [18]. High sensitivity (hs) CRP is also a biomarker of cardiovascular risk in both healthy individuals and those with established CVD, evidence of the inflammatory mechanisms underpinning vascular stiffening and atherosclerotic plaque formation [19,20]. hsCRP levels < 1 mg/L reflect a low systemic inflammatory status and lower atherosclerotic risk; between 1–3 mg/L indicates moderate cardiovascular risk and levels > 3 mg/L indicate higher cardiovascular risk [21].

The present study was based on the adult Cardiovascular Stream of the HHS and aimed to determine whether, 3.5 to 4 years after the smoke event, exposed adults had detectable clinical or subclinical CVD, as reflected by serum levels of hsCRP.

## 2. Materials and Methods

### 2.1. Study Design, Setting and Participants

Data collection for this cross-sectional analysis took place during the period October 2017 to May 2018. Eligible participants were men aged 55–89 years, or women aged 60–89 years, who lived in Morwell (exposed) or Sale (unexposed) at the time of the mine fire, and who had completed the HHS’s baseline Adult Survey [22]. Existing evidence suggests that people with pre-existing cardiovascular conditions may have increased vulnerability to fire-related smoke exposure [23]. Hence those who had reported a cardiovascular condition on the Adult Survey were oversampled such that 50% of those invited had reported a doctor-diagnosis of angina, heart attack, heart failure, irregular heart rhythm/arrhythmia, stroke and/or other cardiovascular disease. Residents from selected areas in Sale were deemed a suitable comparison group to Morwell because they had comparable median age, household size, socio-economic status and population stability to Morwell. CSIRO’s modelling had identified Sale as having minimal smoke exposure during the mine fire event [10].

The Cardiovascular Stream had a target sample size of 330 exposed participants and 165 unexposed participants in order to achieve 80% power to detect a 33% increase in mean C-reactive protein (CRP) between groups. Recruitment was by mailed invitation to a weighted random sample of 1,133 eligible participants, with up to two reminders and follow up phone contact attempted for non-responders. Recruitment continued until target sample sizes were reached.

### 2.2. Outcome and Confounding Variables

Evidence of underlying CVD was determined by serum levels of hsCRP. Other CVD markers were also measured: blood pressure, serum lipids, high sensitivity (hs) troponin, fibrinogen, N-terminal pro B-type natriuretic peptide (NTproBNP), electrocardiographic features suggestive of IHD and endothelial dysfunction as detected by flow-mediated dilatation (FMD).

A sample of blood was collected from a peripheral vein of unfasted participants. hsCRP was measured via the immunoturbidimetric method using an ARCHITECT ci 16,200 analyser [24] (Abbott Laboratories, Chicago, IL, USA). Fibrinogen, hs-troponin, NTproBNP, lipids, glycosylated haemoglobin (HbA_1c_), creatinine and estimated glomerular filtration rate (eGFR) were analysed following standard procedures (see Supplementary Materials Table S1 for laboratory methods).

Height and weight were measured to compute body mass index (BMI) [25]. Blood pressure was measured using an automatic blood pressure monitor with an appropriately sized cuff (Omron, Matsusaka, Japan). Three measurements were recorded at one-minute intervals and the mean of the last two blood pressure measurements was calculated for each participant.

A standard 12-lead electrocardiograph (ECG) was obtained using a portable ECG Machine (Philips TC50; Philips Medical Systems, Andover, MA, USA). Interpretive statements generated by the ECG algorithm were extracted and abnormal traces validated by a cardiologist (SB). The existence of a rhythm abnormality or evidence of underlying IHD was noted, specifically if ECGs demonstrated atrial fibrillation, left bundle branch block, previous infarction, ST depression, T or Q wave abnormalities.

FMD of the right brachial artery was acquired via a Vivid Q ultrasound system (GE Medical Systems, Tirat Carmel, Israel) with 12 MHz linear probe at a standardised room temperature. Participants had not consumed food, caffeine or alcohol for four hours beforehand and smoking and physical activity was documented. Baseline brachial artery images were obtained after 10 min of supine rest. Following five minutes of upper arm cuff occlusion, images were obtained at 30-, 60-, 90-, 120- and 180-s time intervals after cuff deflation. The average diameter (mm) of the brachial artery over six R waves was measured at each interval and deducted from the baseline diameter to obtain the relative change as a percentage of the baseline diameter [26]. The maximal relative change was used in the analysis.

A questionnaire was administered asking about previous diagnoses of cardiovascular conditions, risk factors and demographic information (see Supplementary Materials Table S2). Past history of CVD was defined as a self-reported diagnosis of atrial fibrillation or other arrhythmia, aneurysm, valvular disease, heart failure, myocardial infarction, coronary artery disease, stroke and/or peripheral vascular disease. Alcohol consumption was assessed using the Alcohol Use Disorders Identification Test—Consumption (AUDIT-C) tool, with a score of 0 implying “Non-drinker”, a score of 1–2 for females and 1–3 for males indicating “Low risk” and a score ≥ 3 for females or ≥4 for males considered “high risk” [27,28]. Smoking status was coded as either “Non-smoker” (had not smoked more than 100 cigarettes in entire lifetime), “Current smoker” (had smoked at least 100 cigarettes and was smoking on a regular basis) or “Ex-smoker” (had smoked more than 100 cigarettes in entire lifetime but no longer smoked). Participants engaged in “adequate physical activity” if over the previous seven days, they had done any vigorous physical activity or at least 150 min of moderate physical activity.

Medications were recorded with associated WHO Anatomical Therapeutic Chemical Classification System (ATC) codes [29]; see Supplementary Material Table S3 for a list of ATC codes determined to be antihypertensive medications, lipid-lowering therapy or anti-inflammatory and immunosuppressant medications.

Participants who self-reported a doctor-diagnosis of type 2 diabetes, were taking oral hypoglycaemic medications or insulin, or had a HbA_1c_ ≥ 6.5% were coded as having diabetes mellitus.

Demographic measures included educational attainment and the Index of relative Socio-economic Disadvantage (IRSD), with higher scores indicating less disadvantage [30].

### 2.3. Statistical Analyses

Descriptive statistics were used to compare demographic characteristics and health-related risk factors between exposed and unexposed participants. Weighting methods were used to correct for over sampling of participants with a history of CVD, as well as possible selection bias in the Adult Survey. Weighted Pearson χ^2^ tests were used to report *p*-values for categorical measures and weighted t-tests for continuous measures. When the distribution was skewed, non-parametric weighted Somers’ D statistics were used.

Linear regression was used to compare continuous outcomes between exposed and unexposed participants using mean differences (mean diff) and adjusted mean differences (adj mean diff) controlling for key confounders. Log-transformation was used for the skewed variables hsCRP, NTproBNP and troponin. Differences between exposure groups in outcomes measured as categorical variables were presented as crude and adjusted relative odds ratios (adj OR) using logistic regression incorporating key confounders.

All of the regression analyses accounted for post-stratification and sampling weights, sampling stratification (exposed vs unexposed) and clustering at household level. Statistical analysis and data transformations were performed using Stata version 15 (2015) (Stata Corporation, College Station, TX, USA).

### 2.4. Ethical Considerations

Ethics approval was granted by the Monash University Human Research Ethics Committee (Project Number 1078). Participants signed an informed consent statement and were compensated for their time with a $50 gift card. Abnormal results were communicated, with the participant’s permission, to their regular general practitioner for further management as appropriate.

## 3. Results

### 3.1. Participants

**Figure 1:**
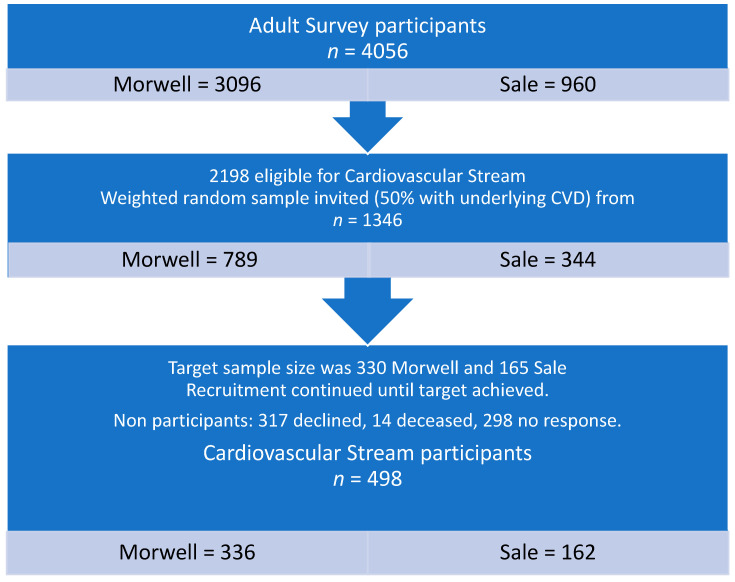
Flow diagram of participant recruitment.

Recruitment closed when a total of 498 participants (37%; 336 exposed and 162 unexposed) had undergone clinical testing (see Figure 1).

Table 1 provides a summary of participant demographic and social characteristics. Exposed Morwell and unexposed Sale participants differed significantly in terms of employment and IRSD score, with a lower score for Morwell indicating greater socioeconomic disadvantage. The exposed group also had a higher proportion of non-Caucasian participants.

Table 2 summarises the clinical characteristics of participants by exposure group. Compared with the unexposed comparison group, exposed participants were less likely to be engaged in adequate physical activity, more likely to be taking lipid-lowering and anti-inflammatory medications and they had a higher mean HbA_1c_.

### 3.2. Outcomes

There was no statistically significant difference in the weighted median of hsCRP between exposed and unexposed participants (Table 3). Unexposed Sale participants demonstrated higher total and low density lipoprotein (LDL) cholesterol levels compared with exposed Morwell participants, while all other biomarkers, ECG and FMD results were similar between the two groups.

As shown in Table 4, univariate and multivariable regression analyses indicated that there was no evidence that exposure group was associated with hsCRP. After adjustment for potential confounders, hsCRP was positively associated with current smoking status, BMI ≥ 25 kg/m^2^ and HbA_1c_. Having a university or other tertiary degree and taking lipid lowering therapy were negatively associated with hsCRP.

Apart from LDL cholesterol, which tended to be higher among unexposed participants, all other markers of CVD were comparable between the two groups, even after adjustment for potential confounders (see Table 5). Additional review of medical conditions which had been self-reported by participants in the Adult Survey one to two years previously identified four participants who had self-reported liver diseases, and 96 exposed participants (weighted mean 28%) and 36 unexposed participants (20%) who had self-reported asthma or chronic obstructive pulmonary disease (COPD); all of which could cause inflammation. Additional analysis (not tabulated) indicated that after exclusion of the four liver disease cases, and after inclusion of the asthma/COPD variable as an additional confounder in the regression models for inflammatory markers, the overall results did not differ.

## 4. Discussion

This latest analysis from the Hazelwood Health Study showed that adults from the community most highly exposed to PM_2.5_ from the mine fire smoke, did not exhibit consistent evidence of clinical or subclinical CVD as measured by serum biomarkers, blood pressure, FMD and ECG approximately four years after the event. However, previous Hazelwood Health Study analyses have shown increased risk of cardiovascular-related deaths in the 6 months after the mine fire [15], increased vascular stiffness in young children 3 years after exposure [16] and an association between mean PM_2.5_ exposure and increased risk of cardiovascular related ambulance attendances in the 3.5 years after the fire [17].

There is a strong evidence base for the causal association between particulate matter air pollution and cardiovascular disease, particularly for long-term exposures [1,12]. A systemic inflammatory response to inhaled particles is believed to be the causal pathway for the effect of PM on the cardiovascular system [31]. This inflammation also likely affects the autonomic nervous system, resulting in arrhythmias, vasoconstriction and platelet activation [2,31]. With persistent inflammation, subclinical cardiovascular disease may develop, possibly leading to acute coronary syndrome, stroke and heart failure [2].

Considering our analysis was conducted four years following the exposure period, it is possible that an acute association between smoke exposure and markers of CVD were missed. Short-term (hours to days) exposures to PM have been associated with increased blood pressure, decreased FMD, and hospitalisation or death from cardiac arrhythmias [32,33,34]. Studies of comparable acute exposure events, such as smoke from forest and peat fires, have been associated with increases in inflammatory markers, emergency department visits for cardio-vascular conditions and out-of-hospital cardiac arrests [35,36,37].

There are less consistent results on the acute effects of PM on serum CRP, however observational studies of longer-term exposures tend to demonstrate positive associations [18]. For example, in a study of 44 adults, aged ≥ 60 years, an interquartile increase in PM_2.5_ of the five-day mean was associated with a 14% (95%CI −5.4 to 37%) increase in CRP for all individuals and an 81% (95%CI 21 to 172%) increase in CRP among those with diabetes, obesity and hypertension [38]. However, PM was probably derived from traffic sources in that study, and participants tended to be older than those in the present analysis. As Li et al. noted in their review of PM air pollution on CRP [39], observational studies tend to utilise aggregated rather than individualised PM exposure data, possibly leading to misclassification bias.

Despite the possibility of a missed acute association in the present analysis, hsCRP remains a well-established biomarker of cardio-vascular risk. A systematic review and meta-analysis of 54 long-term prospective studies involving 160,309 CVD-free participants found that increases in CRP were significantly associated with incident IHD, ischaemic stroke and mortality from both vascular and non-vascular causes [20]. CRP had a positive linear relationship with other conventional cardiovascular risk factors such as age, systolic blood pressure and BMI, and was generally higher among smokers and diabetics. This was confirmed in our analysis, with BMI, HbA_1c_ and smoking being associated with hsCRP. We also found that the use of lipid-lowering therapy and higher levels of education were negatively associated with hsCRP, which have been shown previously [40,41,42].

Sale participants demonstrated higher levels of both total and LDL cholesterol, which is consistent with findings from the Adult Survey, where Sale participants were more likely to report having a diagnosis of high cholesterol [43]. However, a greater proportion of Morwell participants were taking lipid-lowering therapy and anti-inflammatories, medications known to decrease hsCRP, highlighting the importance of adjusting for these variables in the analysis.

The rates of underlying rhythm or ischaemic abnormalities detected by ECG were similar for exposed and unexposed participants. FMD results were also comparable between the two groups, providing further evidence for a lack of association between mine fire exposure and sub-clinical vascular disease in the samples studied, four years subsequent to exposure.

Although not considered a primary cardiovascular outcome, HbA_1c_ was higher among exposed participants (6.1% vs. 5.8%, *p* < 0.0001). While this result is unlikely to be of clinical significance, and is probably reflects other lifestyle factors, exposure to PM has been positively associated with HbA_1c_ and blood glucose levels [44].

Combined, the HHS Hazelinks [15], Early-Life Followup [16], Adult Survey [17] and Cardiovascular Streams aim to provide at least ten years of longitudinal data on the cardiovascular health effects of the six-week PM_2.5_-generating mine fire event. These would complement the 10-year Multi-Ethnic Study of Atherosclerosis and Air Pollution (MESA Air) investigating the relation between CVD and long-term ambient air pollution in a US urban setting [45], and the 10-year Corinthia study investigating the relation between CVD and environmental factors in rural and semi-rural areas of Greece [46] as important longitudinal studies contributing to the field of environmental cardiology.

### Strengths and Limitations

These clinical assessments, conducted approximately four years after the mine fire, build upon previous Hazelwood Health Study investigations of cardiovascular outcomes at earlier time-points. Our results provide evidence for a lack of association between mine fire smoke exposure and markers of CVD years later. Our analysis incorporated sophisticated emissions modelling and we used a range of objective cardiovascular measures to ascertain cardiovascular risk, with minimal differences observed between Morwell and Sale participants. Furthermore, such cardiovascular measures were associated in a predictable fashion with other established markers of cardiovascular risk, such as smoking, BMI and the use of lipid-lowering therapy, supporting the validity of our findings. Exclusion of a small number of participants who had previously reported liver disease, and statistical adjustment for those who had previously reported asthma and/or COPD, all conditions which can cause inflammation, made no difference to the overall findings.

However, the cross-sectional design of this analysis limited our ability to draw causal associations between exposure to mine fire emissions and cardiovascular outcomes, because temporality could not be established. Future linkage with healthcare utilisation datasets, planned by the HHS Hazelinks Stream, may yield ongoing insights into whether there is a higher incidence of acute cardiovascular events among residents of Morwell in future.

Baseline demographic and clinical characteristics demonstrated that the two samples differed significantly on a number of confounding variables, with cardiovascular risk factors being more likely among Morwell participants (eg. lower ISRD score, and less physical activity). This would have biased the results in favour of detecting more CVD in Morwell, which was not observed in our analysis.

Our oversampling of participants with existing cardiovascular conditions may have limited the power of the study to detect a mine-fire effect if the association only occurred in those without existing cardiovascular conditions. Our previous research has estimated that there were 26 cardiovascular-related deaths attributable to the mine fire, in the 6 months after the event [15]. Also, it is possible that mine-fire exposed people with resulting cardiovascular conditions left the Latrobe Valley area after the event. Each of these factors might limit the study’s ability to detect symptoms in the local community four years later.

## 5. Conclusions

We found that adults highly exposed to smoke-related PM_2.5_ from a 6-week duration mine fire, did not exhibit consistent evidence of clinical or subclinical CVD as measured by serum biomarkers, blood pressure, FMD and ECG approximately four years after the event. To our knowledge, this is the first clinical study to examine the potential long-term cardiovascular effects of several weeks-duration exposure to PM_2.5_-generating coal mine fire emissions, despite the fact that a number of coal mine fires are currently burning across the globe, and their incidence is increasing [12]. In addition to providing evidence about the long-term health impacts of coal mine smoke emissions, these findings may be applicable to communities exposed for weeks to the recent mega bushfires, such as those which burned in South Eastern Australia during the 2019–2020 summer season, and in the United States in the 2020 summer.

Considering the increased likelihood of future large-scale landscape fires owing to climate change, this research is important to governments and policy makers when determining an appropriate public health response to similar events in the future. Policy-makers and clinicians should consider the present study in the context of both the broader air pollution literature relating PM_2.5_ exposure to cardiovascular risk and other findings released by the HHS, to inform the public health response to any similar event in the future.

## Figures and Tables

**Table 1:** Demographic and social characteristics of exposed and unexposed participants.

Characteristic	Morwell (Exposed) N = 336		Sale (Unexposed) N = 162		*p*-Value
	*n*	Weighted %	*n*	Weighted %	
**Age Category**					
55–69 years	150	45.9	72	49.1	0.785
70–79	127	35.7	63	32.6	
80+	59	18.4	27	18.3	
**Gender (female)**	150	45.2	70	43	0.618
**Ethnicity (Caucasian/white)**	320	95.8	157	99.1	0.033
**Employment status**					
Employed	50	16.9	41	29.1	0.013
Retired	261	75.2	111	65.8	
Other	25	7.9	8	5.1	
**Highest educational qualification**					
Secondary ≤ year 10	136	40.5	55	32.7	0.288
Secondary years 11 or 12	53	15.7	33	21.1	
Certificate (trade/apprenticeship/technicians)	106	31.9	55	35.3	
University/Tertiary degree	39	11.9	18	10.9	
**IRSD Score**	**Weighted Mean**	**SEM**	**Weighted Mean**	**SEM**	***p*-value**
	867.4	8.5	928.2	4.7	<0.001

Abbreviations: IRSD = Index of Relative Socioeconomic Disadvantage; SEM = Standard error of the mean.

**Table 2:** Clinical characteristics of participants from Morwell and Sale.

Characteristic	Morwell (Exposed) N = 336		Sale (Unexposed) N = 162		*p*-Value
	*n*	Weighted %	*n*	Weighted %	
History of cardiovascular diseases *					
Prior to 2014	113	28.2	62	33	0.559
Since 2014	49	12.6	18	11.8	
**Diabetes ^†^**	84	22.9	29	16.1	0.096
**Smoking status**					
Non-smoker	157	46.4	86	54.1	0.123
Ex-smoker	151	44.6	68	41.4	
Current smoker	28	9	8	4.4	
**Alcohol consumption**					
Non-drinker	87	25	37	23	0.341
Low risk	125	36.3	50	31.1	
High risk	124	38.7	74	46	
**Taking antihypertensive medications**	237	67.8	99	59.7	0.091
**Taking lipid-lowering therapy**	168	47.7	63	37.1	0.038
**Taking anti-inflammatory or immunosuppressant medications**	75	20.6	23	12.7	0.031
**Engaged in adequate physical activity**	150	45.2	98	60.3	0.003
**BMI**					
Underweight/normal	56	17.6	40	24.4	0.105
Overweight	112	33.9	57	36.5	
Obese	168	48.5	65	39.1	
**eGFR**					
<60 mL/min/1.73 m^2^	60	17.1	22	13.5	0.323
**HbA_1c_ (%)**	**Weighted Mean**	**SEM**	**Weighted Mean**	**SEM**	***p*-value**
	6.1	0.1	5.8	0.1	<0.001

* = Includes self-reported doctor-diagnosis of peripheral vascular disease, stroke/transient ischemic attack, coronary artery disease, myocardial infarction, heart failure, valvular disease, aneurysm and/or rhythm abnormality. ^†^ = Self-reported doctor diagnosis of diabetes or HbA_1c_ ≥ 6.5% or taking hypoglycaemic medications. Abbreviations: BMI = body mass index; eGFR = Estimated Glomerular Filtration Rate; HbA_1c_ = glycosylated Haemoglobin; SEM = Standard error of the mean.

**Table 3:** Cardiovascular outcomes for participants from Sale and Morwell.

Outcome Variable	Morwell (Exposed) N = 336		Sale (Unexposed) N = 162		*p*-Value
	Weighted Median	IQR	Weighted Median	IQR	
**Biomarkers**					
hsCRP (mg/L)	1.9	1.0–3.9	1.6	0.8–3.4	0.273 *
Fibrinogen (g/L)	3.6	3.2–4.1	3.5	3.1–4.0	0.406
NTproBNP (ng/L)	99.0	55.0–241.0	100.0	50.0–186.0	0.349 *
Troponin (ng/L)	3.0	2.0–5.0	3.0	2.0–5.0	0.079 *
Total cholesterol (mmol/L)	4.5	3.9–5.2	4.9	4.1–5.4	0.005
HDL (mmol/L)	1.3	1.0–1.6	1.3	1.0–1.5	0.286
LDL (mmol/L)	2.3	1.7–3.0	2.7	2.1–3.2	0.005
Triglycerides (mmol/L)	1.7	1.2–2.4	1.6	1.2–2.3	0.236
**Peak FMD (%) ^†^**	3.8	2.0–5.3	3.6	2.0–5.5	0.999
**Blood pressure**					
Systolic BP (mmHg)	132	120–145	134	126–146	0.059
Diastolic BP (mmHg)	71	64–79	74	67–81	0.059
	***n***	**Weighted %**	**N**	**Weighted %**	***p*-value**
**ECG**					
Rhythm abnormality					
No	229	92.3	102	89.4	0.583
Atrial Fibrillation	17	5.6	12	8.6	
Other	7	2.1	2	2.1	
Evidence of underlying IHD					
Yes	57	15.9	21	10.9	0.120

* = Estimated using nonparametric Somers’ D statistics with sample weighting and clustering included. ^†^ = Includes 379 valid FMD tests for 220 Morwell and 159 Sale participants. Eight low quality test results were excluded. Abbreviations: IQR = inter-quartile range; hsCRP = high sensitivity C-reactive protein; NTproBNP = N-terminal pro B-type natriuretic peptide; HDL = high density lipoprotein; LDL = low density lipoprotein; FMD = flow mediated dilatation; BP = blood pressure; ECG= electrocardiograph; IHD = ischaemic heart disease.

**Table 4:** Results of regression analysis for log-hsCRP.

Predictors	Mean Diff	95% CI	*p*-Value	Adj Mean Diff *	95% CI	*p*-Value
**Exposure group** (Morwell)	0.13	−0.09, 0.35	0.240	0.06	−0.15, 0.27	0.583
**Age** (per 5 years)	0.02	−0.04, 0.09	0.459	0.05	−0.02, 0.13	0.140
**Gender** (Female)	0.06	−0.13, 0.26	0.530	0.13	−0.07, 0.33	0.187
**Employment status** (Employed)	−0.09	−0.34, 0.15	0.455	0.10	−0.16, 0.35	0.457
**Highest educational qualification**						
Secondary ≤ year 10	Ref			Ref		
Secondary years 11 or 12	−0.08	−0.35, 0.19	0.552	0.17	−0.10, 0.43	0.229
Certificate (trade/ apprenticeship/technicians)	−0.04	−0.29, 0.20	0.716	0.08	−0.16, 0.31	0.510
University/Tertiary degree	−0.54	−0.81, −0.28	<0.001	−0.29	−0.57, 0.00	0.046
**Smoking status**						
Non-smoker	Ref			Ref		
Ex-smoker	0.06	−0.15, 0.26	0.601	0.06	−0.15, 0.28	0.572
Current smoker	0.40	0.08, 0.72	0.015	0.52	0.21, 0.84	0.001
**Alcohol consumption**						
Non-drinker	Ref			Ref		
Low risk	−0.05	−0.31, 0.21	0.695	−0.06	−0.30, 0.17	0.590
High risk	−0.13	−0.37, 0.11	0.298	−0.03	−0.26, 0.21	0.818
**Adequate physical activity**	−0.17	−0.37, 0.03	0.093	−0.05	−0.24, 0.15	0.642
**Taking lipid-lowering therapy**	−0.19	−0.38, 0.01	0.058	−0.29	−0.49, −0.08	0.006
**Taking anti-inflammatory or immunosuppressant medications**	0.05	−0.22, 0.33	0.697	−0.02	−0.27, 0.23	0.886
**BMI**						
Underweight/Normal (BMI < 25 kg/m^2^)	Ref			Ref		
Overweight (25 ≤ BMI <3 0)	0.44	0.16, 0.72	0.002	0.54	0.25, 0.83	<0.001
Obese (BMI >30 kg/m^2^)	0.74	0.48, 1.01	<0.001	0.79	0.52, 1.06	<0.001
**HbA_1c_ (%)**	0.13	0.05, 0.21	0.001	0.11	0.04, 0.18	0.004

* = Adjusted for age, gender, history of cardiovascular disease, employment status, education, BMI, smoking status, alcohol consumption, physical activity, HbA_1c_, systolic blood. Abbreviations: Mean Diff = mean difference; Adj Mean Diff = adjusted mean difference; hsCRP = high sensitivity C-reactive protein; BMI = body mass index; HbA_1c_ = glycosylated haemoglobin; Ref = reference variable.

**Table 5:** Results of regression analysis for other cardiovascular outcomes (exposed vs unexposed).

Outcomes	Exposed vs. Unexposed			
	Crude Mean Diff (95% CI)	*p*-Value	Adj Mean Diff * (95% CI)	*p*-Value
**Serum Biomarkers**				
Log NTproBNP	0.06 (−0.14, 0.27)	0.550	0.07 (−0.11, 0.25)	0.451
Log Troponin	0.13 (0.00, 0.27)	0.046	0.09 (−0.03, 0.21)	0.124
Total cholesterol	−0.28 (−0.46, −0.09)	0.004	−0.14 (−0.30, 0.01)	0.070
HDL	−0.04 (−0.12, 0.04)	0.286	0.02 (−0.04, 0.08)	0.539
LDL	−0.28 (−0.45, −0.10)	0.002	−0.15 (−0.30, −0.01)	0.038
Triglycerides	0.11 (−0.08, 0.31)	0.257	0.00 (−0.18, 0.19)	0.975
**Peak FMD (%) ‡**	0.08 (−0.48, 0.64)	0.773	−0.01 (−0.54, 0.52)	0.967
**Blood pressure**				
Systolic BP (mmHg)	−3.28 (−6.68, 0.13)	0.059	−3.75 (−7.23, −0.26)	0.035
Diastolic BP (mmHg)	−2.07 (−4.21, 0.08)	0.059	−1.74 (−3.80, 0.31)	0.097
	**Crude OR (95% CI)**	***p*-value**	**Adj OR † (95% CI)**	***p*-value**
**ECG**				
Atrial Fibrillation	0.68 (0.31, 1.52)	0.350	0.41 (0.14, 1.16)	0.094
Other rhythm abnormality	0.75 (0.36, 1.56)	0.442	0.50 (0.20, 1.23)	0.130
Evidence of underlying IHD	1.55 (0.89, 2.69)	0.122	1.52 (0.83, 2.78)	0.177

* = Adjusted for age, gender, employment, education, history of cardiovascular diseases, body mass index, smoking and drinking status, physical activity, HbA_1c_, systolic blood pressure (not including analysis of systolic and diastolic blood pressure), taking lipid-lowering therapy and anti-inflammatory/immunosuppressants; † = Adjusted for age, gender, employment, education, body mass index, smoking and drinking status, physical activity, HbA1c, taking lipid-lowering therapy and taking anti-inflammatory/immunosuppressants; ‡ = Includes 379 valid FMD tests for 220 Morwell and 159 Sale participants. Eight low quality test results were excluded. Abbreviations: Diff = difference; Adj Mean Diff = adjusted mean difference; NTproBNP = N-terminal pro B-type natriuretic peptide; HDL = high-density lipoprotein; LDL = low-density lipoprotein; BP = blood pressure; Adj OR = adjusted Odds Ratio; ECG = electrocardiograph; IHD = ischaemic heart disease.

## Data Availability

Restrictions apply to the availability of these data. Data were obtained from participants and are available from the authors with the permission of the Victorian Department of Health.

## Supplementary Materials

The following are available online at https://www.mdpi.com/1660-4601/18/4/1587/s1, Table S1: Laboratory Methods; Table S2: Cardiovascular questionnaire; Table S3: Medications and corresponding ATC Codes used in the analysis.
