# Supplementary material for: Markers of Cardiovascular Disease among Adults Exposed to Smoke from the Hazelwood Coal Mine Fire
Source: Int J Environ Res Public Health. 2021 Feb 8;18(4):1587. doi: 10.3390/ijerph18041587 (PMC7914645; doi:10.3390/ijerph18041587)
Supplement: Supplementary file 1 [file ijerph-18-01587-s001.pdf]

Markers of cardiovascular disease among adults exposed to smoke from the Hazelwood coal mine fire.

Supplementary Material

Table S1 Laboratory Methods

**Clinical Biochemistry Methods**		
**Test Name**	**Method**	**Instrument**
High sensitivity C-reactive protein	Immunoturbidimetric	Abbott Archicentre ci 16200
Creatinine, enzymatic	Enzymatic	
Cholesterol	Enzymatic	
Triglycerides	Glycerol Phosphate Oxidase	
Cholesterol, high-density lipoprotein (HDL)	Accelerator Selective Detergent	
Cholesterol, low-density lipoprotein (LDL)	LDL was not measured directly but calculated using the Friedewald formula: LDLC = (total CHOL) – (HDLC) – (TG/2.2). This formula cannot be used where TG > 4.5 mmol/L.	
Estimated glomerular filtration rate (eGFR)	eGFR was not measured directly but was calculated using the creatinine result, gender and age as per the CKD-EPI formula	
High sensitivity troponin I	Chemiluminescent Microparticle Immunoassay	
N-terminal pro B-type natriuretic peptide	Electrochemiluminescence Immunoassay	Roche e411
Haemoglobin A1c	Boranate Affiniy HPLC	Trinity Premier Hb9210
**Haematology Methods**		
**Test Name**	**Method**	**Instrument**
Fibrinogen	Von Clauss	Stago STA-R Max2

Table S2 Cardiovascular questionnaire

1. Have you ever been told by a doctor that you have any of the following conditions?	
High blood pressure / hypertension	Yes/No
If yes, year of diagnosis or first episode:	
High blood cholesterol	Yes/No
If yes, year of diagnosis or first episode:	
Atrial Fibrillation	Yes/No
If yes, year of diagnosis or first episode:	
Apart from Atrial Fibrillation, any other irregular heart beat / arrhythmia / palpitation. E.g. heart is out of its normal rhythm, skipped a beat, added a beat, is 'fluttering', or is beating too fast	Yes/No
If yes, year of diagnosis or first episode:	
Anuerysm ie. a localised bulge of a blood vessel, caused by disease or weakening of the vessel wall	Yes/No
If yes, year of diagnosis or first episode:	
Heart valve disease ie. damage or defect in one of the heart valves	Yes/No
If yes, year of diagnosis or first episode:	
Heart failure ie. heart is failing to pump sufficient blood throughout the body	Yes/No
If yes, year of diagnosis or first episode:	
Heart attack / Myocardial infarction	Yes/No
If yes, year of diagnosis or first episode:	
Coronary artery disease or atherosclerosis ie. build-up of plaque in the artery wall that can lead to blockage of the artery	Yes/No
If yes, year of diagnosis or first episode:	
Stroke / Mini stroke / TIA ie. interruption of blood flow to the brain	Yes/No
If yes, year of diagnosis or first episode:	
Vascular disease ie. restriction of blood flow to the arms, legs, hands, or feet.	Yes/No
If yes, year of diagnosis or first episode:	
If yes, please specify_________________	
Diabetes type 2 ie. usually called non-insulin-dependent, or adult-onset	Yes/No
If yes, year of diagnosis or first episode:	
To properly evaluate your heart health it is important to know about your family history.<br><br>2. Did any of your close relatives suffer from any of the following conditions/events before the age of 60 years? A close relative includes: parents, brothers, sisters.	
Heart attack / Myocardial infarction	Yes/Don't know/No
If yes, do you know the estimate age at first diagnosis?	Yes/No
If yes, estimate age at first diagnosis:	

Coronary artery disease or atherosclerosis ie. build-up of plaque in the artery wall that can lead to blockage of the artery	Yes/Don't know/No
If yes, do you know the estimate age at first diagnosis?	Yes/No
If yes, estimate age at first diagnosis:	
Stroke / Mini stroke / TIA ie. Interruption of blood flow to the brain	Yes/Don't know/No
If yes, do you know the estimate age at first diagnosis?	Yes/No
If yes, estimate age at first diagnosis:	
Vascular disease ie. restriction of blood flow to the arms, legs, hands or feet.	Yes/Don't know/No
If yes, please specify ____________	
If yes, do you know the estimate age at first diagnosis?	Yes/No
If yes, estimate age at first diagnosis:	
Diabetes type 2 ie. usually called non-insulin-dependent, or adult-onset	Yes/Don't know/No
If yes, do you know the estimate age at first diagnosis?	Yes/No
If yes, estimate age at first diagnosis:	
To properly evaluate your cardiovascular health it is important to know about your smoking history and other exposure to smoke	
3. Have you smoked at least 100 cigarettes, or a similar amount of tobacco, in your entire lifetime?	Yes/No
If yes, which one of the following best describes your smoking status?	o You currently smoke daily?<br>o You currently smoke at least weekly, but not daily?<br>o You currently smoke less often than weekly?<br>o You don't smoke now but you used to?
At which age did you last stop smoking?	
For how many years in total have you smoked? (if stopped and started, add smoking periods together)	
Over those years, what is the average number of cigarettes that you have smoked per day, or if less than daily, per week or month?	
Please state period (per day, per week, or per month)	o Per day<br>o Per week<br>o Per month
4. Have you been exposed to tobacco smoke on most days and nights in the last 12 months?	Yes/No
If yes, Not counting yourself, how many people in your household smoke regularly?	
Where do they usually smoke?	o Inside the house

	○ Outside the house<br>○ Both inside and outside the house
Because alcohol use can affect health and interfere with certain medications and treatments, it is important that we ask you questions about your use of alcohol. Your answers will remain confidential, so please be as accurate as possible. Try to answer the questions in terms of 'standard drinks'.	
5. How often did you have a drink containing alcohol in the past year?	○ Never → move to question 6<br>○ Monthly or less<br>○ 2 to 4 times per month<br>○ 2 to 3 times per week<br>○ 4 or more times per week
How many drinks did you have on a typical day when you were drinking in the past year?	○ 1 or 2<br>○ 3 or 4<br>○ 5 or 6<br>○ 7 to 9<br>○ 10 or more
How often did you have 6 or more drinks on one occasion during the past year?	○ Never<br>○ Less than monthly<br>○ Monthly<br>○ Weekly<br>○ Daily or almost daily
To properly evaluate your heart, it is important to know about your physical activity. The questions start with vigorous activity and end with sitting. The first question is about vigorous activity. Vigorous activity requires a large amount of effort and causes rapid breathing and a large increase in your heart rate. (Examples: running, fast cycling, aerobics, fast swimming, heavy shovelling or digging ditches, carrying / moving heavy loads (>20kg))	
6. During the last 7 days, did you do any vigorous physical activity for at least 10 minutes at a time?	Yes/ No
If yes, how many days per week did you do vigorous activity?	
These questions are about moderate activity. Moderate activity requires a moderate amount of effort and noticeably increases your heart rate. (examples: brisk walking, dancing, gardening, housework or domestic chores, active involvement in games and sports with children/walking domestic animals, general building tasks (eg roofing, painting), carrying / moving moderate loads (<20kg)	
7. During the last 7 days, did you do any moderate physical activity for at least 10 minutes at a time?	Yes/No
If yes, how many days per week did you do moderate activity?	

How many minutes per day did you do moderate activity?	
The next questions are about walking (examples: walking to the shops or to the end of the street)	
8. During the last 7 days, did you do any walking for at least 10 minutes at a time?	Yes/No
If yes, how many days per week did you walk for at least 10 minutes?	
How many minutes per day did you walk for at least 10 minutes at a time?	
This question is about activities you did over the 7 days while sitting or lying down. Don't count the time you spent in bed. For each of the activities only count the time when this was your main activity. For examples if you are watching television and doing a crossword, count it as television time or crossword time but not as both.	
9. During the last 7 days, how much time in total did you spend sitting or lying down and …	
Watching television or videos/DVDs	
Using the computer / internet	
Reading	
Doing hobbies, e.g. craft, crosswords	
Socializing with friends or family	
Driving or riding in a car, or time on public transport	
10. Which if these best describes you current employment status?	o Self employed<br>o Employed full-time<br>o Employed part-time or casual<br>o Unemployed<br>o A student<br>o Engaged in home duties<br>o Retired<br>o Unable to work<br>o Other (please specify ________________)
11. Which ethnic (ancestry) group do you consider yourself to be part of?	o Caucasian/ White<br>o Aboriginal / Torres Strait Islander<br>o Asian<br>o Polynesian/Melanesian/Maori<br>o North African / Middle Eastern<br>o Black African<br>o Central / South American<br>o Other (please specify)________________)

Table S3 Medications and corresponding ATC Codes used in the analysis

Medication Group	ATC Codes
**Antihypertensive medications**	
Diuretics	Thiazides: C03EA14, C03BA04, C03BA03, C03AA03, C03BA11, C03BA08, C03BA02, C03BA10. Loop diuretics: C03CA02, C03CX01, C03CA01, C03CA03, C03CA04.
Beta blocking agents	Selective: C07AB04, C07AB03, C07AB05, C07AB07, C07AB08, C07AB02, C07AB12, C07AB13. Non-selective: C07AA01, C07AA19, C07AA30, C07AA15, C07AA14, C07AA18, C07AA12, C07AA02, C07AA23, C07AA03, C07AA05. Alpha and beta blocking agents: C07AG02.
Angiotensin converting enzyme inhibitors	C09AA07, C09AA01, C09AA08, C09AA02, C09AA09, C09AA03, C09AA13, C09AA04, C09AA06, C09AA05, C09AA11,C09AA10
Angiotensin II antagonists	C09CA06, C09CA02, C09CA04, C09CA01, C09CA07, C09CA03.
Selective calcium channel blockers	Dihydropyridine derivatives: C08CA01, C08CA02, C08CA03, C08CA09, C08CA13, C08CA04, C08CA05, C08CA10, C08CA07, C08CA08. Benzothiazepine derivatives: C08DB01. Phenylalkylamine derivatives: C08DA02, C08DA01.
Alpha-adrenoreceptor antagonists	C02CA07, C02CA04, C02CA01, C02CA08.
Anti-SNS agents	C02AC01, C02CC07, C02AC02, C02AB01, C02AC05.
Arteriolar vasodilators	C04AX37, C02DB01, C02DC01.
Other blood pressure lowering agents	C03BX03.
Combination drugs	Diuretics and potassium-sparing agents: C03EA01, C03EA06, C03EA12, C03EA14, C03EA15, C03EA21, C03EA41, C03EB01, C03EB02, C03EB21. Beta blocking agents and diuretics: C07BA01, C07BA02, C07BA05, C07BA12, C07BA14, C07BA18, C07BB02, C07BB03, C07BB04, C07BB07, C07BG02, C07CA02, C07CA03, C07CA05, C07CA23, C07CB02, C07CB03, C07CB04, C07CB08, C07DA05, C07DB01. Loop diuretics and ACE inhibitors: C09BA01, C09BA02, C09BA03, C09BA04, C09BA05, C09BA06, C09BA07, C09BA08, C09BA09, C09BA13. Thiazides and angiotensin II antagonists: C09DA01, C09DA03, C09DA04, C09DA06, C09DA07. ACE inhibitors and calcium channel blockers: C09BB05, C09BB10. Calcium channel blockers and diuretics: C08GA01, C08GA02. Angiotensin II antagonists & Calcium channel blockers C09DB01, C09DB02, C09DB04. Angiotensin II antagonists, other combinations: C09DX01, C09DX03

**Lipid modifying agents**	HMG CoA reductase inhibitors: C10AA
**Anti-inflammatory OR immunosuppressant medications**	
Corticosteroids for systemic use	H02 (all)
Antiinflammatory and antirheumatic products	Non-steroids: M01A (all). In combination with corticosteroids: M01BA
Hormone replacement therapy	Estrogens: GO3C. Progestogens G03D. Progestogens and estrogens in combination G03FA, G03FB.
Aspirin	B01AC06
